# KMT2D Deficiency Causes Sensorineural Hearing Loss in Mice and Humans

**DOI:** 10.3390/genes15010048

**Published:** 2023-12-28

**Authors:** Allison J. Kalinousky, Teresa R. Luperchio, Katrina M. Schrode, Jacqueline R. Harris, Li Zhang, Valerie B. DeLeon, Jill A. Fahrner, Amanda M. Lauer, Hans T. Bjornsson

**Affiliations:** 1 McKusick-Nathans Department of Genetic Medicine, Johns Hopkins School of Medicine, Baltimore, MD 21205, USA; akalino1@jhmi.edu (A.J.K.); teresaluperchio@gmail.com (T.R.L.); harrisjac@kennedykrieger.org (J.R.H.); lzhang32@jhmi.edu (L.Z.); jfahrne1@jhmi.edu (J.A.F.); 2 Department of Otolaryngology-Head and Neck Surgery and Center for Hearing and Balance, Johns Hopkins School of Medicine, Baltimore, MD 21205, USA; katrinaschrode@cdrewu.edu (K.M.S.); alauer2@jhmi.edu (A.M.L.); 3 Department of Neurology, Kennedy Krieger Institute, Baltimore, MD 21205, USA; 4 Department of Anthropology, University of Florida, Gainesville, FL 32610, USA; vdeleon@ufl.edu; 5 Department of Pediatrics, Johns Hopkins School of Medicine, Baltimore, MD 21205, USA; 6 Landspitali University Hospital, 102 Reykjavik, Iceland; 7 Faculty of Medicine, University of Iceland, 101 Reykjavik, Iceland

**Keywords:** Kabuki syndrome, congenital hearing loss, genetic syndrome, hair cells, MLL2

## Abstract

Individuals with Kabuki syndrome type 1 (KS1) often have hearing loss recognized in middle childhood. Current clinical dogma suggests that this phenotype is caused by frequent infections due to the immune deficiency in KS1 and/or secondary to structural abnormalities of the ear. To clarify some aspects of hearing loss, we collected information on hearing status from 21 individuals with KS1 and found that individuals have both sensorineural and conductive hearing loss, with the average age of presentation being 7 years. Our data suggest that while ear infections and structural abnormalities contribute to the observed hearing loss, these factors do not explain all loss. Using a KS1 mouse model, we found hearing abnormalities from hearing onset, as indicated by auditory brainstem response measurements. In contrast to mouse and human data for CHARGE syndrome, a disorder possessing overlapping clinical features with KS and a well-known cause of hearing loss and structural inner ear abnormalities, there are no apparent structural abnormalities of the cochlea in KS1 mice. The KS1 mice also display diminished distortion product otoacoustic emission levels, which suggests outer hair cell dysfunction. Combining these findings, our data suggests that KMT2D dysfunction causes sensorineural hearing loss compounded with external factors, such as infection.

## 1. Introduction

Mendelian disorders of the epigenetic machinery (MDEM) are a class of disorders caused by germline mutations in the genes encoding cellular components that maintain epigenetic modifications [1]. Most of these epigenetic components have broad roles in development [1,2] and are known to affect gene expression [3]; therefore, individuals with an MDEM often have multisystemic features [1]. One of these disorders is Kabuki syndrome type 1 (KS1), which is caused by pathogenic variants in *KMT2D*, a gene encoding a histone methyltransferase [4] responsible for maintaining histone H3K4 methylation, and thus promoting open chromatin [5,6]. The loss of KMT2D has been shown to disrupt DNA methylation levels in peripheral blood in individuals with KS1 [7,8,9], including genes that are expressed in the inner ear, such as *Myo1F*; however, whether this disrupted chromatin state plays a mechanistic role in the disease is uncertain. In fact, *KMT2D* is relatively highly expressed in most tissues [10], and the mechanistic basis of cell-type-specific disease manifestations in KS1 is currently unknown. 

The cardinal features of KS reported in the literature include mild to moderate intellectual disability, characteristic facial features, persistent fetal fingerpads, postnatal growth deficiency, and skeletal anomalies [11,12]. In addition to these phenotypes, another clinically relevant and penetrant phenotype in humans with KS is progressive hearing loss, with estimate frequencies of individuals with KS with hearing loss ranging from 24 to 65% [13,14]. This frequency is higher than what is seen in the general population, with estimates of approximately 1% of newborns presenting with hearing loss from birth [15] and approximately 17–20.3% of individuals by the age of 12 experiencing unilateral loss [15,16]. This prevalent phenotype of KS, however, has received less attention in recent years. 

Current clinical dogma states that hearing loss in KS is primarily conductive, resulting from repetitive ear infections in early life in the context of immune dysfunction [17] or related to the abnormal craniofacial structure [18]. Like many features in KS, the presence and severity of hearing loss appears to vary among individuals, and more interestingly, there are examples of monozygotic twins discordant for hearing loss [19]. Ear malformations and deafness are one of the criteria for diagnosing CHARGE syndrome, another MDEM [20] often mistaken for KS in the clinic [21,22,23]. KS and CHARGE have overlapping DNA methylation gene targets [8], which could explain some of the clinical overlap seen between the two syndromes. Despite relatively little research into the hearing loss phenotype of KS, individuals with KS are recommended to have annual hearing evaluations. However, there are currently no clinical recommendations or treatments to prevent or mitigate the progressive hearing loss, and the mechanistic basis is currently unknown. Hearing at birth appears to be in the normal range for a large portion of individuals with KS, in contrast to some other causes of pediatric hearing loss [24].

In this study, we collect clinical data on participants with KS1 to clarify the prevalence, severity, and onset timing of hearing loss. By performing focused studies in a *Kmt2d*-deficient mouse model, which demonstrates many features observed in humans including neuronal dysfunction [25], growth retardation [18], craniofacial defects [18], and immune dysregulation [17], we are able to measure hearing function from early developmental stages and assess structural changes [26]. We demonstrate early-age sensorineural hearing problems in the mouse model of KS1, suggesting that, despite the previously reported late onset, this disease phenotype may be determined in prenatal life. 

## 2. Materials and Methods

### 2.1. Mice

*Kmt2d^+/βGeo^* mice have been previously described [25]. Specifically, genotyping was performed using the following primers: β-GeoF-(CAAATGGCGATTACCGTTGA) and β-GeoR-(TGCCCAGTCATAGCCGAATA), which are specific for the targeted allele. We also used TcrdF-(CAAATGTTGCTTGTCTGGTG) and TcrdR-(GTCAGTCGAGTGCACAGTTT) as controls to ensure a sufficient DNA concentration. Mice for longitudinal studies were group-housed up to five per cage in ventilated racks and kept in a quiet, low-traffic room in order to minimize any extraneous noise exposure. They were provided a standard rodent diet and access to filtered water via an automatic watering system. All experiments were performed using mouse protocols approved by the Animal Care and Use Committee of the Johns Hopkins University School of Medicine. The mouse protocols used for this study follow the guidelines used by the National Institutes of Health (NIH) for mouse care and handling. 

### 2.2. Auditory Brainstem Response and Distortion Product Otoacoustic Emission Procedure 

Hearing loss in mice was determined using auditory brainstem response (ABR) testing. Procedures were similar to those described by Schrode et al. [27]. Mice were immobilized by brief sedation with 100 mg/kg ketamine and 10 mg/kg xylazine and placed on a heating pad inside a sound-attenuating chamber lined with Sonex acoustic foam in order to reduce acoustic reflections. Body temperature was monitored using a rectal probe to minimize risk of hypothermia. The mice were placed 30 cm from the front of a speaker, with subcutaneous electrodes placed on the skull. 

ABR stimuli consisted of single brief clicks that excite a large number of auditory neurons at a wide range of frequencies (click, 8, 12, 16, 24, and 32 kHz) to assess the overall integrity of auditory function. These frequencies activate distinct regions of the cochlea (i.e., base, mid-turn, and apex) in order to determine frequency-specific cochlear functioning. Volume levels ranged from 10 decibels (dB) sound pressure level (SPL) to 100 dB SPL, which corresponds to sounds ranging from quiet ambient noises to the sound of a jack hammer 1 meter away. ABR thresholds were determined as the lowest recognizable ABR response, or at which ABR peak-to-peak amplitude was two standard deviations above the average background noise. Lack of response was indicated as a value of 100 dB SPL. 

Following ABR measurement, distortion product otoacoustic emissions (DPOAEs) were recorded at nine weeks of age. For DPOAEs, mice were sedated and placed in a sound-attenuating chamber, as used for ABR testing. A small probe containing two speakers and a microphone was inserted into the ear canal (ER10C; Etymotic Research). Two tones were presented through the probe, where the ratio of the frequencies was equal to 1.2, and f1 was 10 dB higher than f2. We tested frequencies (f2) of 8, 12, 16, and 18 kHz at sound levels up to a maximum of 65 dB SPL (f1). The distortion product was measured at the frequency corresponding to 2f1-f2. The noise floor was determined as the level of the response at adjacent frequencies.

### 2.3. Statistical Analysis 

The thresholds from the ABR and the DPOAE measurements, and the proportion of missing hair cells were analyzed using either a mixed-effect analysis, with the mouse type (i.e., wildtype (WT) or KS1) as a between-group variable, and threshold at different frequencies or at specific time points from neonatal to adult as the repeated measure variable, or a two-way analysis of variance (ANOVA). The cochlear length and height, and the inner ear volume of the KS1 and WT mice were compared using a two-tailed unpaired t-test. These analyses were performed using GraphPad Prism version 10.1.1 for Mac OS X, GraphPad Software, Boston, MA USA, www.graphpad.com (accessed on 5 July 2022). A *p*-value of 0.05 or lower was considered significant.

### 2.4. Immunofluorescence 

Immunofluorescent staining was performed as previously described by Schrode et al. [27]. Briefly, transcardial perfusions were performed and the cochleae were extracted and fixed for an hour. Cochleae were then decalcified in 1% EDTA, and then the organ of Corti was dissected into 5–6 pieces. The cochlear pieces were placed in a blocking buffer of 5% normal goat serum, 10% bovine serum albumin, and 0.5% Triton X-100 for one hour, then incubated overnight at 4 degrees Celsius in mouse anti-SV2 (1:500, DSHB cat# SV2, RRID:AB_2315387) and rabbit polyclonal anti-myosin 6 (1:500, Sigma-Aldrich, Saint Louis, MO, USA, cat# M5187, RRID:AB_260563) in half-concentration blocking buffer. The next day, cochlear pieces were rinsed, incubated in secondary antibodies of goat anti-mouse AF488 (1:1000, Thermo Fisher Scientific, cat# A-10667, RRID:AB_2534057, Waltham, MA, USA) and goat anti-rabbit AF568 (1:1000, Thermo Fisher Scientific, cat# A-11036, RRID:AB_10563566) in half concentration blocking buffer for two hours at room temperature. The pieces were then rinsed again, mounted in Fluoromount g (Southern Biotech, Birmingham, AL, USA) on subbed slides, and coverslipped. Inner and outer hair cells were manually quantified using Fiji [28] at nine frequency locations along the cochlea, falling at half-octave intervals between 4 and 64 kHz.

### 2.5. Volumetric Measurements 

Aspects of this work have been previously described by Fahrner et al. [18]. Briefly, skulls from six-week-old mice were fixed in 4% paraformaldehyde, washed, and then transferred to 70% ethanol. A desktop microtomographic imaging system (Skyscan 1172, Bruker, Billerica, MA, USA) was used to obtain high-resolution images. This work was performed following the recommendations of the American Society for Bone and Mineral Research (ASBMR) [29]. Scans were collected using 79–80 kVp and 124–125 uA parameters, and image volumes were reconstructed as 8-bit DICOM data with cubic voxels of 0.027974 millimeters on each side. Three-dimensional models of the negative space of the inner ear were isolated using VG StudioMax software 3.0 (Volume Graphics) and a density threshold of 50 on a 0–225 scale. Additional voxels were added as necessary to create continuity along the semicircular canals. Cochlear height was measured as the distance from the apex of the cochlea to the posterior point on the saccule. Cochlear length was measured as the curvilinear distance of landmarks placed in the groove between scala vestibuli and scala tympani from the helicotrema to the base of the saccule.

### 2.6. Participants

To better understand the prevalence and onset of hearing loss in KS1, we gathered information from individuals with KS1 and their families using a questionnaire. We recruited 21 children and adults with a molecularly confirmed diagnosis of KS1 from the Epigenetics and Chromatin clinic at the Johns Hopkins Hospital, as well as those who previously consented for research unrelated to hearing loss and agreed to be contacted about relevant research studies. All participants carry pathogenic or likely pathogenic variants by ACMG-AMP criteria. Written responses and/or interviews to assist with the questionnaire about hearing loss were completed by all participants. We asked when hearing loss was formally diagnosed, the severity of the hearing loss, as well as any contributing factors that might relate to hearing loss. We specifically asked if the patient experienced frequent ear infections, as current clinical dogma suggests that a frequent cause of hearing loss in KS is a result of frequent ear infections. We also asked questions regarding if the patient passed their newborn screen, if their hearing loss has become worse over time, if any immediate or distant family members have hearing loss, if they have any structural abnormalities, if they had been exposed to any known ototoxic medications, if they had surgeries performed by an ENT, and if they experience any pain or ringing in their ears. This study was approved by the Johns Hopkins Medical Institutions Institutional Review Board, and all participants underwent a written informed consent process. Deidentified participant information can be found in Table 1.

## 3. Results

### 3.1. Individuals with KS1 Have Multiple Factors Contributing to Hearing Loss

**Figure 1:**
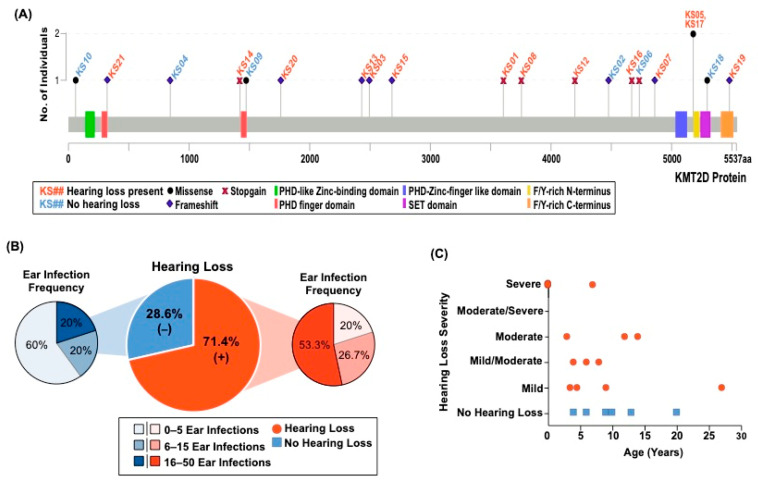
Hearing loss is a common phenotype in individuals with KS1. (**A**) Distribution of participants’ variants (*n* = 21) across the KMT2D protein domains. The type of variant is indicated by the symbol on the graph, with the height of each point representing the number of individuals with that specific variant. KS05 and KS17 are unrelated individuals. Only coding variants of the *KMT2D* gene are shown. All coordinates are in genome build hg38. (**B**) Pie chart displaying those with hearing loss (orange) and those without hearing loss (blue). The smaller pie charts show the breakdown of the frequency of ear infections (high, moderate, and mild). (**C**) Individuals with KS1 without hearing loss are represented in blue, plotted with their current age. The orange dots represent KS1 individuals with hearing loss, plotted with their age of onset of loss (*x*-axis), and the severity of hearing loss (*y*-axis).

We recruited 21 individuals with KS1, nine of whom are male (42.86%) and 12 of whom are female (57.14%), with ages ranging from one year and eight months to 36 years of age. Figure 1A shows the distribution of variants throughout the *KMT2D* gene, with 71.42% having a truncating variant, and the remaining having a missense variant. 

In our cohort, 15 participants (71.43%) reported current hearing loss, with the average age of hearing loss onset occurring at seven years of age. All 12 females had hearing loss whereas only three of the nine males (33.33%) had hearing loss. Three participants had hearing loss from birth. One participant reported hearing loss at two years of age and used hearing aids from age seven to nine; however, currently at the age of 13, the participant no longer reports hearing loss and did not indicate any intervention. In terms of number of ear infections, 53.33% of those with hearing loss experienced a high number of infections, which we have defined as ranging from 16 to 50 ear infections. Interestingly, 40% of those without hearing loss experienced either a high number of infections or a moderate number of ear infections, which we have defined as 6 to 15 ear infections (Figure 1B). Four individuals (19.05%) reported having outer, middle, and/or inner ear abnormalities (i.e., abnormal ear canals, malformed cochlea, or the need for a prosthetic stapes). Notably, both the individual with a malformed cochlea, and another individual with an absent cranial eighth nerve (auditory nerve) presented at birth with hearing loss. Two of the six individuals (33.33%) without hearing loss are under the age of seven years, which we found as the average age that hearing loss presented, and one of those six individuals was utilizing hearing aids at seven years of age but no longer reports hearing loss (Figure 1C). Of the 15 individuals with hearing loss, 10 reported the type of hearing loss diagnosis, with one having conductive hearing loss, six having sensorineural hearing loss, and three having mixed hearing loss. These data support the notion that hearing loss is a common, but variable, phenotype in KS1. Although these data are unable to clarify the etiology of the hearing loss in KS1, they suggest a multi-factorial causation. 

### 3.2. Kmt2d^+/βGeo^ Mice Have Early Hearing Impairment

To investigate whether mice with *Kmt2d* mutations demonstrate hearing loss, we performed ABRs on a previously established mouse model of KS1 (*Kmt2d^+/βGeo^* mice). ABRs were first performed on a small cohort (*n* = 8: KS1 = 4, WT (*Kmt2d^+/+^*) = 4) of adult male mice (8 weeks of age), using a variety of frequencies (8, 12, 16, 24, and 32 kHz) to rapidly assess hearing loss. We observed a significant difference in thresholds (*p* = 0.0130) between KS1 mice and WT littermates (Figure S1).

**Figure 2:**
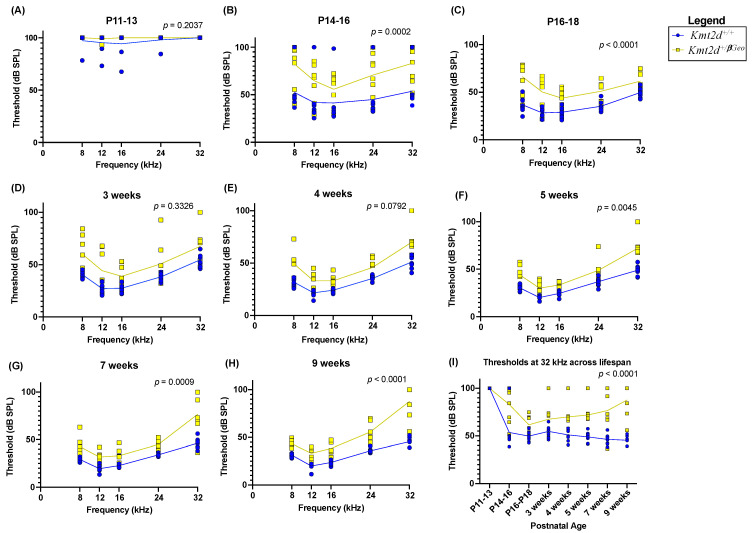
KS1 mice (*Kmt2d^+/βGeo^*) display significant abnormalities of hearing from the onset of hearing in comparison to wildtype (WT) littermates. Plots of threshold (dBSPL) vs. tone frequency (kHZ) as measured by auditory brainstem response (ABR) testing at (**A**) P11-13 (*p* = 0.2037), at (**B**) P14-16 (*p* = 0.0002), at (**C**) P16-18 (*p* < 0.0001), at (**D**) 3 weeks (*p* = 0.3326), at (**E**) 4 weeks (*p* = 0.0792), at (**F**) 5 weeks (*p* = 0.0045), at (**G**) 7 weeks (*p* = 0.0009), and at (**H**) 9 weeks of age (*p* < 0.0001). Blue represents WT mice (*n* = 8) and yellow represents KS1 mice (*n* = 8). (**I**) Plot of threshold vs. postnatal age at a frequency of 32 kHz (*p* < 0.0001). Two-way ANOVA (**A**–**H**) or mixed-effects analysis (**I**) were used.

Following this pilot experiment, ABRs were then performed on KS1 mice (*n* = 8) and WT littermates (*n* = 8) from the onset of hearing (approximately postnatal day 12 (P12)) through early adulthood (nine weeks), to determine when KS1 mice present with hearing loss. Performing a two-way ANOVA, we found no difference between the thresholds found for KS1 and WT mice at P11-P13 across all frequencies (*p* = 0.2037), which is expected as mice are born with no response to airborne sound and begin to show measurable ABRs around P12 [30]. Unexpectedly, we observed significant abnormalities of hearing in KS1 mice at all frequencies immediately from the onset of hearing at P14-P16 (*p* = 0.0002), and at P16-P18 (*p* < 0.0001), at five weeks (*p* = 0.0045), at seven weeks (*p* = 0.0009), and at nine weeks (*p* < 0.0001) (Figure 2A–H). At three weeks, one KS1 mouse died and was replaced with another KS1 mouse of the same age. At four weeks, one WT mouse and two KS1 mice died, and were replaced with one WT mouse and one KS1 mouse of same age. This result contradicts current clinical dogma that hearing loss onset is later in life for individuals with KS. Additionally, the cochlea is tonotopically organized such that individual segments along the length of the cochlea respond to different frequencies; thus, the fact that all frequencies are affected supports the hypothesis that the whole cochlea is affected. No difference was found in thresholds between sexes for WT or KS1 mice. 

### 3.3. Hearing Loss in Kmt2d^+/βGeo^ Mice Is Exacerbated with Age

In addition to the early onset hearing loss, we observed that hearing loss increased drastically with age at higher frequencies. Loss of sensitivity at high frequencies is common in age-related hearing loss and noise-induced damage to the cochlea, as higher frequencies are detected by the vulnerable base of the cochlea where sound enters from the middle ear. At 32kHz, the highest frequency tested, hearing sensitivity decreases with age (*p* < 0.0001), and at nine weeks, the oldest age tested, many of the KS1 mice are approaching deafness (Figure 2I). The average threshold recorded at nine weeks of age in KS1 mice was 87.69 DB SPL, while the WT mice had an average threshold of 45.44 DB SPL. A second independent cohort showed a significant difference in thresholds between KS1 mice (*n* = 7) and WT littermates (*n* = 7) at seven (*p* = 0.0076) and eight weeks (*p* = 0.0029). This cohort suggests a trending loss but does not reach a significant difference at three through six weeks of age, which can support the variable phenotype seen in individuals with KS1 (Figure S2A–F). To note, for this cohort, at four weeks, one KS1 and one WT mouse was added, and at five weeks, one WT mouse died. This progressive loss is similar to the acquired hearing loss observed in some individuals with KS, which anecdotally occurs in middle childhood. 

### 3.4. Kmt2d^+/βGeo^ Mice Have No Obvious Structural Abnormalities in the Inner Ear

**Figure 3:**
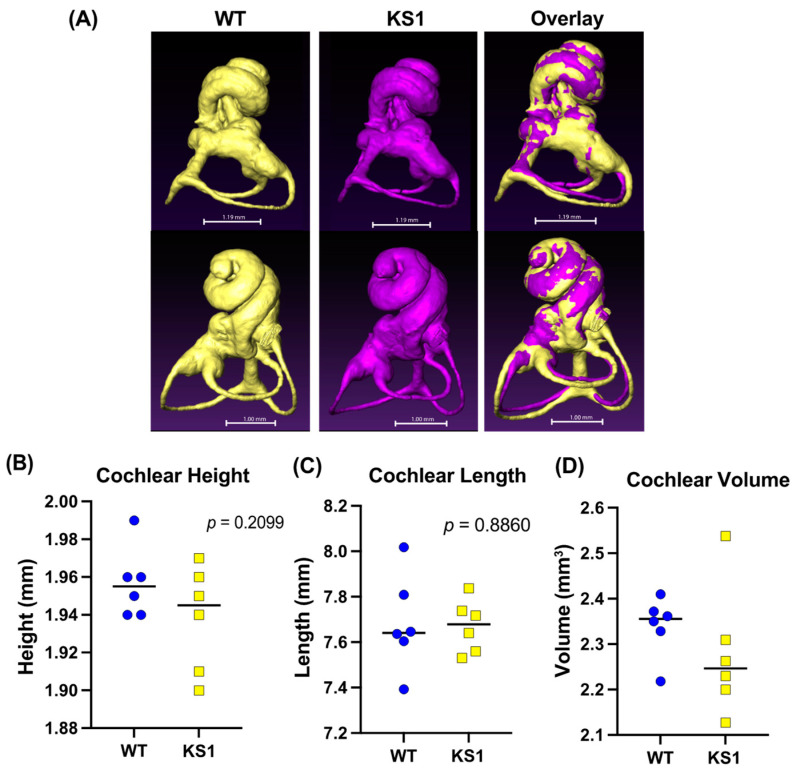
KS1 mice show no significant structural differences of the inner ear compared to wildtype (WT) littermates. (**A**) Representative 3D reconstructions of the inner ear of KS1 mice (*n* = 6) and WT littermates (*n* = 6). Overlay shows no signs of gross structural differences between genotypes. (**B**) Plot of cochlear length of WT and KS1 mice. No significant difference was found between genotypes (*p* = 0.2099). (**C**) Plot of cochlear length of WT and KS1 mice. No significant difference was found between genotypes (*p* = 0.8860). (**D**) Plot of cochlear volume of WT and KS1 mice. No significant difference was found between genotypes (*p* = 0.3508). Blue represents WT mice and yellow represents KS1 mice. Two-tailed unpaired *t*-tests were used.

To test whether our mice had inner ear abnormalities similar to those seen in CHARGE syndrome (for example the commonly reported Mondini dysplasia—reduced length and number of cochlear turns), we performed microcomputed tomography (micro-CT) imaging and created 3D reconstructions of the inner ear in both *Kmt2d^+/βGeo^* mice (*n* = 6) and WT littermates (*n* = 6) (Figure 3A). These images did not suggest any gross structural abnormalities and, more specifically, both the cochlea and the semicircular canals appeared normal. Cochlear height (*p* = 0.2099) (Figure 3B) and length (*p* = 0.8860) (Figure 3C), and inner ear volume (*p* = 0.3508) (Figure 3D) showed no statistically significant difference between *Kmt2d^+/βGeo^* mice and WT littermates. Additionally, features associated with Mondini dysplasia did not appear within the KS1 mice samples. 

### 3.5. Kmt2d^+/βGeo^ Mice Display Diminished DPOAE Levels

**Figure 4:**
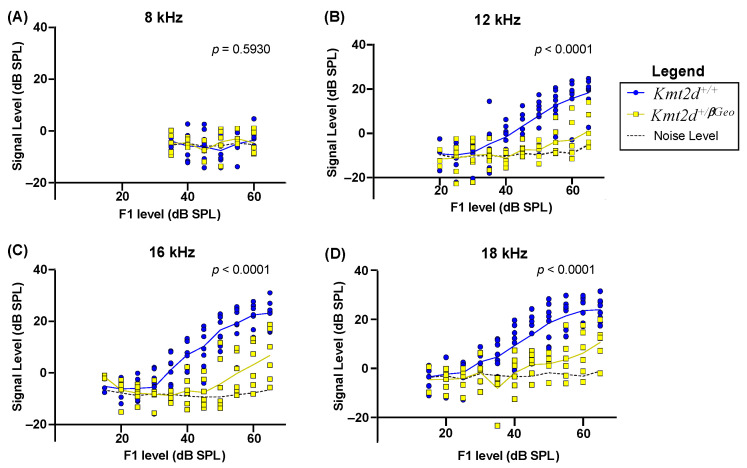
KS1 mice (*Kmt2d^+/βGeo^*) display diminished distortion product otoacoustic emission (DPOAE) levels in comparison to WT littermates. Plot of signal level (dB SPL) as a function of stimulus sound level (dB SPL) for stimuli at (**A**) 8 kHz (*p* = 0.5930), (**B**) 12 kHz (*p* < 0.0001), (**C**) 16 kHz (*p* < 0.0001), and (**D**) 18 kHz (*p* < 0.0001). Blue represents WT mice (*n* = 8) and yellow represents KS1 mice (*n* = 6). The dashed black line represents the average noise level of the DPOAE. Mixed-effect analyses were used.

DPOAEs are generated within the cochlea in response to pairs of tone stimuli. These responses are linked to outer hair cell (OHC) integrity, as OHCs play an essential role in cochlear amplification [31]. Here, we tested four frequencies (8, 12, 16, and 18 kHz) presented at a range of sound levels (15–65 dB SPL) in a cohort of KS1 (*n* = 6) and WT mice (*n* = 8). Using a mixed-effect analysis, we found that the KS1 mice displayed significantly diminished signal levels (amplitude of the DPOAE response, in dB SPL) in comparison to WT littermates at all frequencies (*p* < 0.0001) except 8 kHz (Figure 4A–D), indicating OHC dysfunction is present in KS1 mice. 

### 3.6. Outer Hair Cells Have Subtle Damage in Adult Kmt2d^+/βGeo^ Mice

As the gross physical structure of the vestibulocochlear organ seemed to be normal, we asked if there were any abnormalities that could be ascertained at the histological level. The cochlea contains a row of inner hair cells (IHCs) which receive auditory input, and three rows of OHCs which provide nonlinear amplification that improves sensitivity and frequency selectivity. IHCs are innervated by auditory nerve fibers, and this connection transduces auditory stimulus (vibrations) into neural signals sent to the central auditory system. Both IHCs and OHCs receive descending inputs from olivocochlear efferent neurons which serve to modulate cochlear activity, primarily through inhibition. Examination of the sensory epithelium by immunofluorescence and staining of key hair cell markers in a small cohort showed a subtle increase in the percent of missing OHCs in adolescent (nine-week-old) *Kmt2d^+/βGeo^* mice (*n* = 2) compared to WT littermates (*n* = 3) at mid-to-high frequencies (>5.6) (Figure S3A–C). While the differences in OHCs did not reach statistical significance due to the small cohort size, combining this finding with the diminished DPOAEs, we believe that the damaged/missing OHCs in the KS1 mice may be representative of what you would expect in a larger cohort. 

## 4. Discussion

It is reported that hearing loss is common in individuals with KS [13,14], with the thought that frequent ear infections resulting from immune dysfunction [17] and/or structural abnormalities of the ear [18] contribute to this loss. In contrast to many other causes of dominant hearing loss [32], most individuals with KS have normal hearing at birth. We have found that nearly two-thirds of individuals with KS1 have hearing loss, with the average age of presentation being seven years. While ear infections and structural abnormalities seem to contribute to the hearing loss seen in our cohort, our data suggest that there may be additional causes. Forty percent of participants with hearing loss reported sensorineural hearing loss, which can result from damage to the hair cells within the inner ear, the vestibulocochlear nerve, or the brain’s central processing centers. We also find a sex difference, with all females reporting hearing loss and only one-third of males reporting hearing loss. Interestingly, three of the five individuals with a missense variant reported no hearing loss, while the remaining two individuals reporting hearing loss are unrelated individuals possessing the same variant. While this would need to be examined in a larger cohort, hearing loss could be another phenotype that has a milder presentation in individuals with KS with missense variants. 

The average age of onset of hearing loss in our human cohort was seven years of age, and, interestingly, one-third of the individuals without hearing loss were younger than seven years. Additionally, one individual had hearing loss and utilized hearing aids from age seven to age nine; however, this individual no longer reports hearing loss and was counted in the no-hearing-loss category. Therefore, it is possible that the frequency of hearing loss in our cohort may be an underestimate as these younger individuals may still develop hearing loss as they age. Additionally, substantial cochlear damage can occur without a large effect to behavioral thresholds, but will appear on physiological responses (i.e., ABRs and central auditory processing (CAP)) [26,33,34]. It is possible that those without hearing loss may have only had behavioral hearing assessments, which would not detect early signs of cochlear dysfunction. Periodic ABR or auditory steady-state response (ASSR) screening may be useful for individuals with KS. 

Our mouse model of KS1 was found to have hearing impairment from the onset of hearing, with hearing worsening to near complete deafness at high frequencies in a cohort followed over time. Current anecdotal evidence suggests that the hearing loss is secondary to infection; however, we did not observe any obvious signs of infections in the mice. More in-depth investigation of the inner ear of the mice is required to further answer this question, but based on observing hearing deficiency at the onset of hearing, we do not expect ear infections are the primary factor contributing to the hearing loss in the KS1 mice. We did not test hearing loss in a KS type 2 (KS2) mouse model, which does not have IgA dysfunction [35]; however, hearing loss is observed in individuals with KS2 [36,37]. This early hearing impairment and progressive hearing loss observed in our mouse model of KS1 can instead show that early KMT2D-dysfunction, likely prenatal, may manifest in postnatal life and predispose the subject to hearing loss. If cochlea abnormality are developmentally acquired, this will decrease the window of opportunity for the treatment of this potential outcome measure. With treatments on the horizon, the early detection of hearing loss and an understanding of this therapeutic window is important for improving the quality of life of individuals with KS. 

As longitudinal ABR measures require repeated sound exposure, it is possible this contributed to the hearing loss of the mice, as mice predisposed to hearing loss may be more susceptible to the louder sounds used for testing. Additionally, it has been published that a mouse model of noise-induced hearing loss had increased ABR thresholds and loss of outer hair cells [38]. This repeated sound exposure would need further investigation in KS1 mice, but, if true, could lead to recommendations that those with KS are particularly careful of protecting against sound exposures. As some individuals with KS use headphones to isolate from environmental sounds, usage with loud volumes may mean they are at risk of further damaging their hearing. 

Additionally, our data showed that adult KS1 mice displayed diminished DPOAEs at a variety of frequencies, indicating that the KS1 mice have damaged and/or missing outer hair cells. When staining key hair cell markers, we find a proportion of outer hair cells missing in the KS1 mice; however, this finding is limited by the small sample size. Further quantification of stereocilia tip links (filaments connecting stereocilia) would need to be performed to make any strong conclusions on contribution of OHCs to the hearing loss seen in the KS1 mice. These tip links are notoriously delicate and are often damaged during cochlear dissections, so this analysis was not performed here. 

Unlike mouse models of CHARGE syndrome, our mice did not appear to have inner-ear structural abnormalities despite a shared clinical phenotype between syndromes. We did, however, find that approximately 19% of participants with KS1 had outer, middle, and/or inner ear structural abnormalities. This finding does support clinical dogma that some of the hearing loss seen in individuals with KS is a result of a craniofacial structural abnormality; however, this only explains a small proportion of the hearing loss seen in our cohort. By combining the data gathered from participants with KS1 and from the KS1 mouse model, our findings suggest that KMT2D-dysfunction predisposes individuals to early and progressive hearing loss, with other external factors, such as infection, adding to this hearing loss.

## 5. Conclusions

Hearing loss is common in KS1, multifactorial in origin, and can be acquired at any time. Hearing screening should be offered to patients as part of general care, but further studies are required to establish the mechanistic basis of hearing loss in KS1.

## Figures and Tables

**Table 1:** Survey data from participants with disease causing variants in *KMT2D*.

ID	Variant	Age	Hearing Loss/Type	Age of Hearing Loss	Severity	Total # of Ear Infections	Outer, Middle, and/or Inner Ear Abnormalities
KS01	c.10813C>T	22	+/Bilateral	14	Moderate	2	-
KS02	c.13432dupC	20	-	NA	NA	Many	-
KS03	c.7481_7482insT	20	+/Bilateral	4	Mild/moderate	20+	+
KS04	c.2533delC	9	-	NA	NA	3	-
KS05	c.15536G>A	30	+/Unilateral	27	Mild	~16	-
KS06	c.14194C>T	4	-	NA	NA	1	+
KS07	c.14580dupT	36	+/Bilateral	6	Mild/moderate	~40	-
KS08	c.11263C>T	13	+/Bilateral	7	Mild	12	-
KS09	c.4421G>T	10	-	NA	NA	9	-
KS10	c.175A>G	6	-	NA	NA	1	-
KS11	IVS50+5G>A	15	+/Unilateral	12	Moderate	~10	NR
KS12	c.12592C>T	21	+/Bilateral	8	Mild/moderate	20	+
KS13	c.7291_7294delTCTG	17	+/Bilateral	At Birth	Profound	1	+
KS14	c.4265G>A	18	+/Bilateral	7	Right-severe. Left consistent.	30	-
KS15	c.8045_8046delAG	3	+/Bilateral	At Birth	Left- moderate/severe Right-severe	10+	-
KS16	c.14006C>G	29	+/Bilateral	3.5	Mild	25	-
KS17	c.15536G>A	28	+/Bilateral	4.5	15–30 decibel hearing loss	~50	-
KS18	c.15884G>C	13	-/Bilateral	2	Normal now but significant at 2 years of age	8–9	-
KS19	c.16437delT	15	+/Bilateral	3	Right-moderate. Left-mild/moderate	25+	-
KS20	c.5278_5279delAA	12	+/Unilateral	9	Mild	8	-
KS21	c.970dupC	1.7	+/Bilateral	At birth	Profound	0	-

NA, not applicable; NR, not reported.

## Data Availability

Data are contained within the article and Supplementary Materials.

## Supplementary Materials

The following supporting information can be downloaded at https://www.mdpi.com/article/10.3390/genes15010048/s1. Figure S1: A preliminary cohort of KS1 male mice, aged eight weeks, show significant abnormalities of hearing (*p* = 0.0130) in comparison to WT male littermates; Figure S2: A second independent KS1 cohort displays a trend of hearing impairment at younger ages, with statistically significant differences in thresholds between KS1 and WT littermates at older ages; Figure S3: KS1 mice show subtle increase in percent missing of outer hair cells compared to WT littermates.
