# Supplementary material for: KMT2D Deficiency Causes Sensorineural Hearing Loss in Mice and Humans
Source: Genes (Basel). 2023 Dec 28;15(1):48. doi: 10.3390/genes15010048 (PMC10815913; doi:10.3390/genes15010048)
Supplement: Supplementary file 1 [file genes-15-00048-s001.zip › genes-2784693-supplementary.pdf]

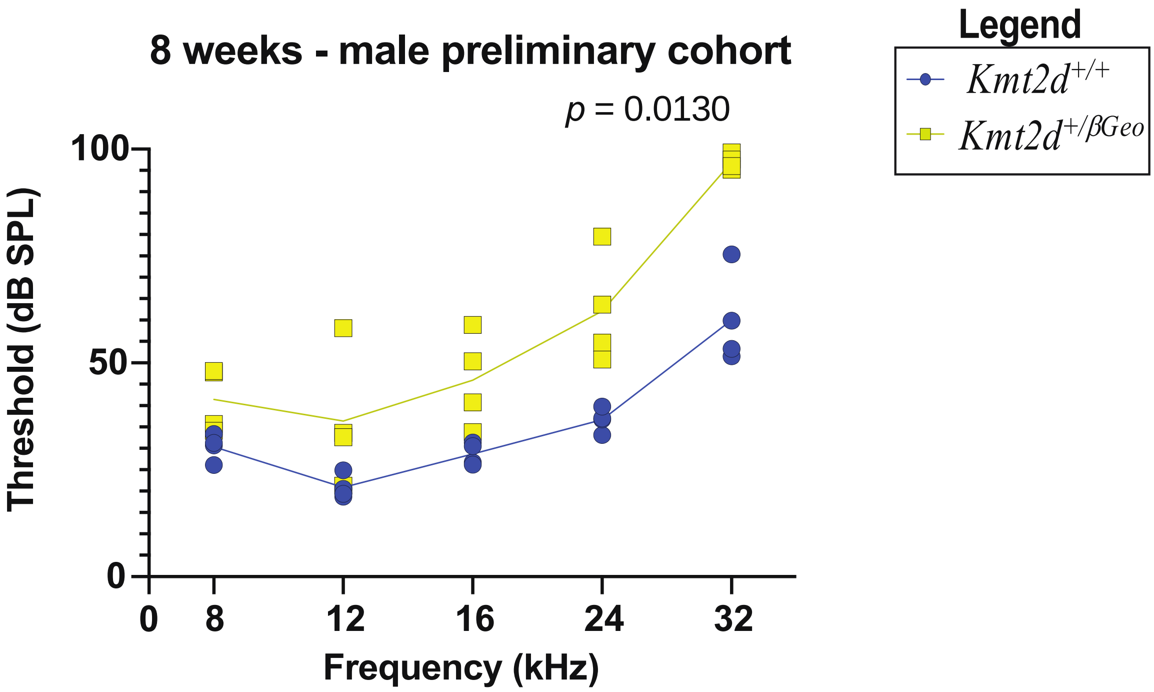

Figure S1. A preliminary cohort of KS1 male mice, aged eight weeks, show significant abnormalities of hearing ($p = 0.0130$) in comparison to wildtype (WT) male littermates. Blue represents WT mice ($n = 4$) and yellow represents KS1 mice ($n = 4$). Two-way ANOVA was used.

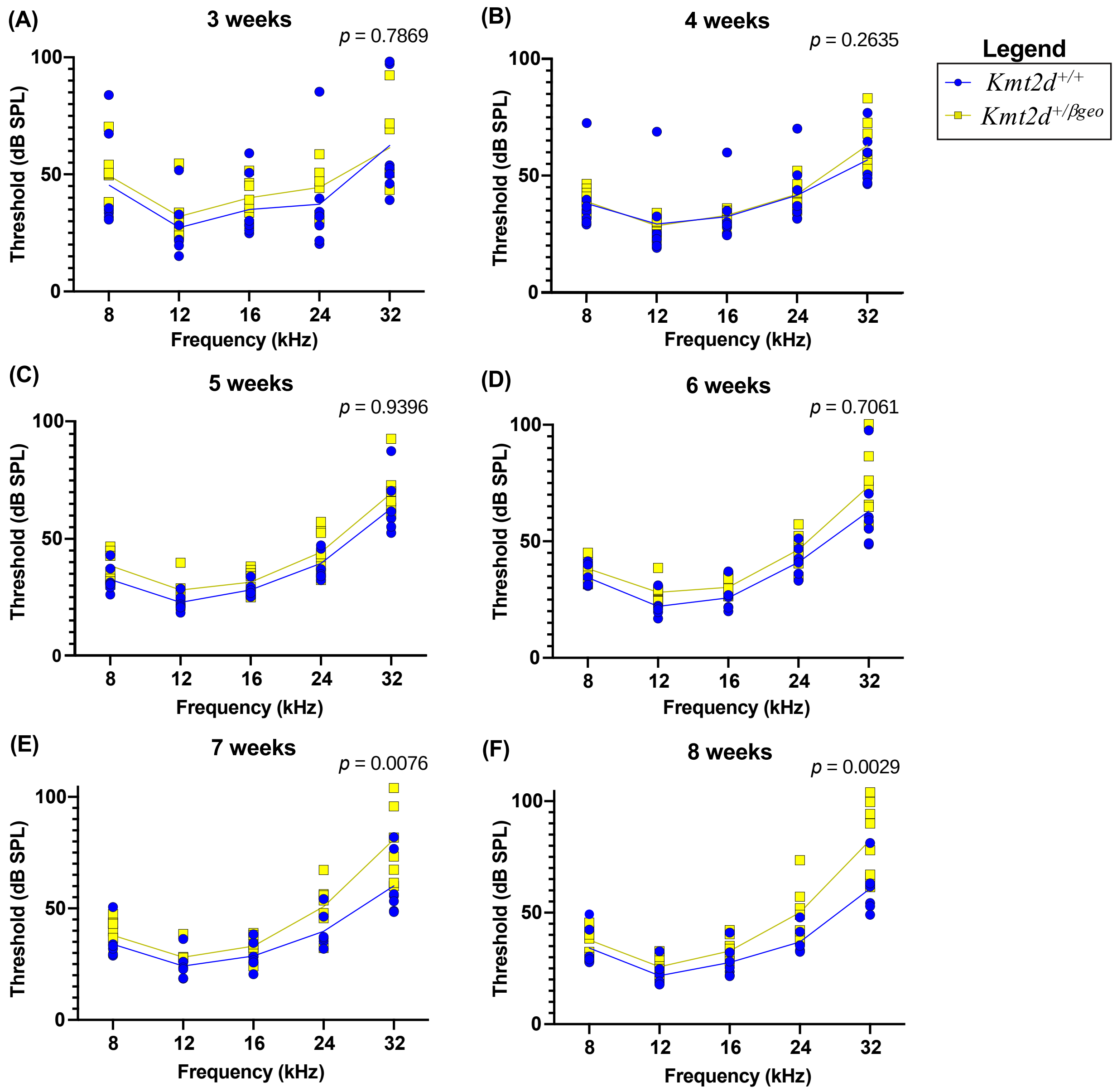

Figure S2. A second independent KS1 cohort displays a trend of hearing impairment at younger ages, with statistically significant differences in thresholds between KS1 and wildtype (WT) littermates at older ages. Plot of threshold (dBSPL) vs tone frequency as measured by ABR testing at **(A)** 3 weeks (p = 0.7869), **(B)** 4 weeks (p = 0.2635), **(C)** 5 weeks (p = 0.9396), **(D)** 6 weeks (p = 0.7061), **(E)** 7 weeks (p = 0.0076), and **(F)** 8 weeks of age (p = 0.0029). Blue represents WT mice (n = 7) and yellow represents KS1 mice (n = 7). Two-way ANOVA were used.

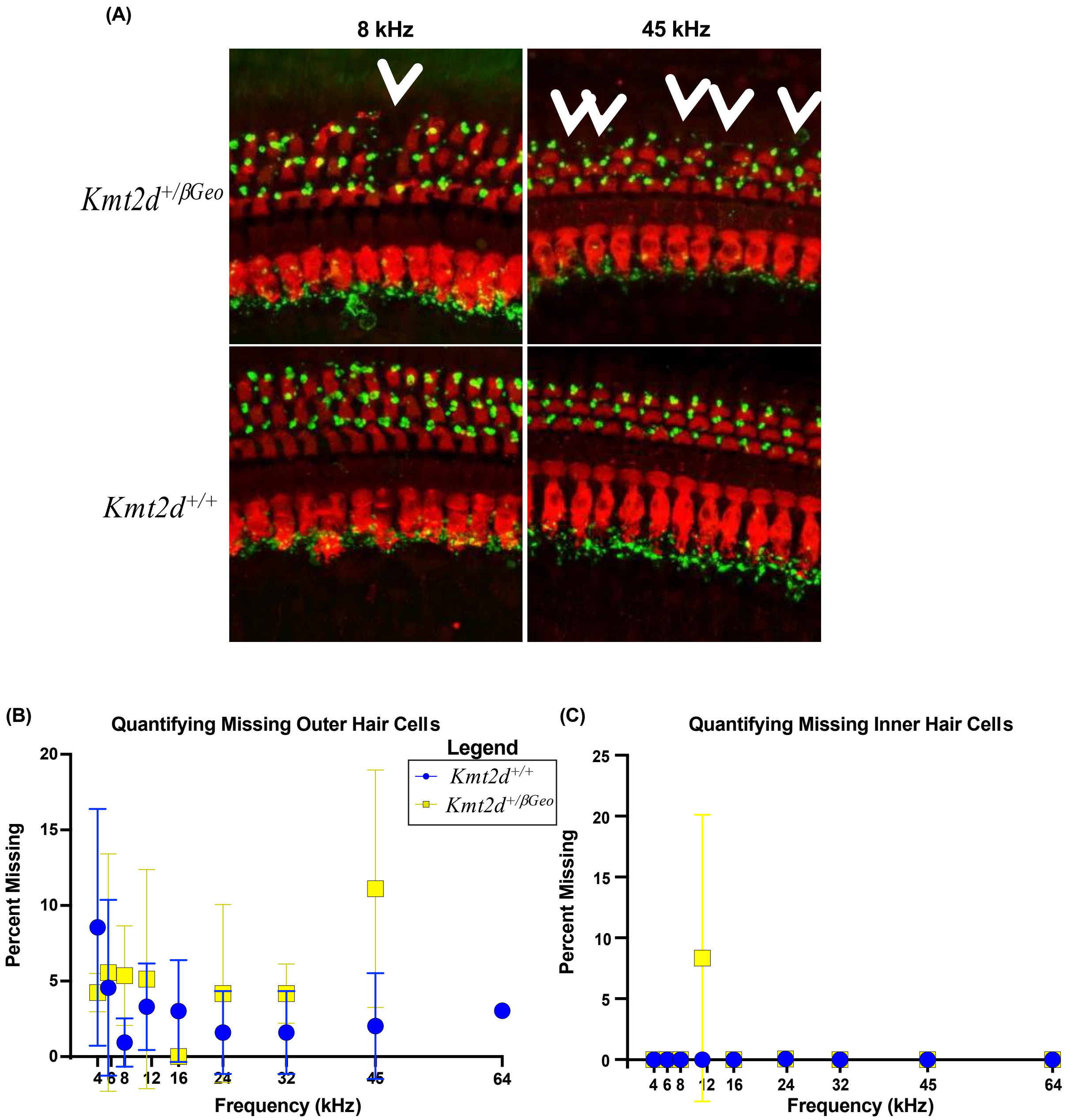

Figure S3. KS1 mice show subtle increase in percent missing of outer hair cells compared to wildtype (WT) littermates. **(A)** Plot of percent missing of outer hair cells (identified using immunostaining) at varying frequencies in the cochlea. **(B)** Plot of percent missing of inner hair cells at varying frequencies in the cochlea. KS1 mice (n = 2) are shown in yellow and WT mice (n = 3) are shown in blue.
